# MicroRNA-144 acts as a tumor suppressor by targeting Rho-associated coiled-coil containing protein kinase 1 in osteosarcoma cells

**DOI:** 10.3892/mmr.2021.12344

**Published:** 2021-08-05

**Authors:** Jianjun Huang, Ying Shi, Hui Li, Meisongzhu Yang, Guohong Liu

Mol Med Rep 12: 4554-4559, 2015; DOI: 10.3892/mmr.2015.3937

Following the publication of this paper, it was drawn to the Editors’ attention by a concerned reader that certain of the Transwell cell migration data shown in Figs. 2C and D, and 4B and D, were strikingly similar to data appearing in different form in other articles by different authors. Owing to the fact that the contentious data in the above article had already been published elsewhere, or were already under consideration for publication, prior to its submission to *Molecular Medicine Reports*, the Editor has decided that this paper should be retracted from the Journal. The authors were asked for an explanation to account for these concerns, but the Editorial Office did not receive any reply. The Editor apologizes to the readership for any inconvenience caused.

